# Subclinical cardiac impairment relates to traditional pulmonary function test parameters and lung volume as derived from whole-body MRI in a population-based cohort study

**DOI:** 10.1038/s41598-021-95655-7

**Published:** 2021-08-09

**Authors:** Ricarda von Krüchten, Roberto Lorbeer, Christopher Schuppert, Corinna Storz, Blerim Mujaj, Holger Schulz, Hans-Ulrich Kauczor, Annette Peters, Fabian Bamberg, Stefan Karrasch, Christopher L. Schlett

**Affiliations:** 1 Department of Diagnostic and Interventional Radiology, Medical Center, University of Freiburg, Hugstetter Straße 55, 79106 Freiburg, Germany; 2 Department of Radiology, Ludwig-Maximilians-University Hospital, Marchioninistraße 15, 81377 Munich, Germany; 3 Department of Diagnostic and Interventional Radiology, University Hospital of Heidelberg, Im Neuenheimer Feld 110, 69120 Heidelberg, Germany; 4 Department of Neuroradiology, Medical Center, University of Freiburg, Breisacher Straße 64, 79106 Freiburg, Germany; 5 Institute of Epidemiology, Helmholtz Zentrum München, German Research Center for Environmental Health, Ingolstädter Landstraße 1, 85764 Neuherberg, Germany; 6 Translational Lung Research Center Heidelberg, Member of the German Center of Lung Research (DZL), Im Neuenheimer Feld 156, 69120 Heidelberg, Germany; 7 Institute and Outpatient Clinic for Occupational, Social and Environmental Medicine, Inner City Clinic, Ludwig-Maximilians-University Hospital, Ziemssenstraße 1, 80336 Munich, Germany; 8 Member of the German Center for Lung Research (DZL), Comprehensive Pneumology Center Munich (CPC-M), Max-Lebsche-Platz 31, 81377 Munich, Germany

**Keywords:** Epidemiology, Magnetic resonance imaging, Whole body imaging

## Abstract

To evaluate the relationship of cardiac function, including time-volume-curves, with lung volumes derived from pulmonary function tests (PFT) and MRI in subjects without cardiovascular diseases. 216 subjects underwent whole-body MRI and spirometry as part of the KORA-FF4 cohort study. Lung volumes derived semi-automatically using an in-house algorithm. Forced expiratory volume in one second (FEV1), forced vital capacity (FVC), and residual volume were measured. Cardiac parameters derived from Cine-SSFP-sequence using cvi42, while left ventricle (LV) time-volume-curves were evaluated using pyHeart. Linear regression analyses assessed the relationships of cardiac parameters with PFT and MRI-based lung volumes. Mean age was 56.3 ± 9.2 years (57% males). LV and right ventricular (RV) end-diastolic-, end-systolic-, stroke volume, LV peak ejection- and early/late diastolic filling rate were associated with FEV1, FVC, and residual volume (excluding late diastolic filling rate with FEV1, LV end-systolic/stroke volume and RV end-diastolic/end-systolic volumes with residual volume). In contrast, LV end-diastolic volume (ß = − 0.14, p = 0.01), early diastolic filling rate (ß = − 0.11, p = 0.04), and LV/RV stroke volume (ß = − 0.14, p = 0.01; ß = − 0.11, p = 0.01) were inversely associated with MRI-based lung volume. Subclinical cardiac impairment was associated with reduced FEV1, FVC, and residual volume. Cardiac parameters decreased with increasing MRI-based lung volume contrasting the results of PFT.

## Introduction

Chronic respiratory diseases, such as chronic obstructive pulmonary disease (COPD) including emphysema, are among the leading causes of morbidity and mortality^1^, claiming some 2.5 million lives worldwide in 2015^2^. Moreover, chronic respiratory diseases increase the cardiac overload and may result in cardiac impairment, thus increasing morbidity and further affecting quality of life^3^. Therapeutic options for chronic respiratory disease and consequent cardiac impairment are limited and increase health care costs. Therefore, early detection and management are important measures to slow COPD progression, exacerbations, and hospitalizations^4^.

Recent studies reported on the association of impaired cardiac function and maladaptive deformation with respiratory parameters. Watz et al. showed decreased cardiac chamber sizes and impaired left ventricular diastolic filling patterns as well as global right-heart dysfunction in patients with known hyperinflation^5^. Another study of cardiac volumes assessed by magnetic resonance imaging (MRI) in patients with severe emphysema showed reduced left and right ventricle end-diastolic volumes, suggesting an association with hyperinflation^6^. Larger cohorts such as the Multi-Ethnic Study of Atherosclerosis (MESA), which used computed tomography (CT) for assessment of the lung volume as well as MR imaging for cardiac assessment, further characterized the relationship between cardiac impairment and COPD. In this study within MESA, Barr et al. observed that emphysema severity was linearly related to impaired left ventricular filling, reduced stroke volume, and lower cardiac output, but without change in the ejection fraction^7^. While most studies refer to diastolic dysfunction assessed by increased left-ventricular (LV) end-diastolic volume, the right ventricular (RV) function was assessed in the same MESA study. Pulmonary hyperinflation was associated with smaller RV end-diastolic volume, stroke volume, cardiac output, and reduced RV mass. An increase in RV afterload was observed among current smokers^8^, potentially related to diminished pulmonary vascular bed due to apoptosis of pulmonary endothelium^9^, or endothelial dysfunction^10^, as well as an increase in pulmonary vascular resistance, among others. However, cardiac time-volume-curves have so far not been assessed in this context. The diastolic function can be more precisely characterized by measuring early and late ventricular diastolic filling rates. Cardiovascular magnetic imaging can result in a separate volume calcualtion at each phase, and the filling rate curve (dV/dT) at different parts of the cycle can evaluate diastolic function^11^. Detection of subclinical cardiac impairment, especially in the early stages of COPD, is key to reduce disease burden. Melerba et al. reported LV diastolic dysfunction using Doppler-echocardiography in patients with early-stage COPD and with no clinical signs of cardiovascular dysfunction^12^. Moreover, Thomson et al. reported that reduced forced expiratory volume in one second (FEV1) and forced vital capacity (FVC) by spirometry were associated with smaller ventricular volumes and reduced ventricular mass determined by cardiovascular MRI^13^.

While pulmonary functional tests (PFT) are widely available for evaluating respiratory function, advanced imaging by radiation-free visualization techniques, such as whole-body MRI are also now accessible. For research purposes, whole-body MRI may represent a promising imaging technique that enables cardiac and pulmonary assessment at the same time^14^. Recently, our KORA (Cooperative Health Research in the Region of Augsburg) cohort study suggested that lung volume derived from whole-body MRI is associated with PFT-derived residual volume, but also with the FEV1/FVC ratio (Tiffeneau index), as well as with a clinical history of COPD^15^. Based on this evidence, we explored the clinical value of MRI-derived lung volumes based on a short, non-dedicated T1-Dixon sequence, within the framework of the KORA MRI study, which excluded subjects with known cardiovascular disease. As the first study to evaluate cardiac parameters and lung volumes from one single whole-body MRI scan, we aimed firstly to assess the association of MRI-based cardiac parameters with FEV1, FVC, and residual volume as derived from PFT, and secondly to evaluate the relationship between these cardiac parameters and MRI-derived lung volume.

## Methods

### Study population

The KORA FF4 cohort study represents a broad sample from a general population in the region of Augsburg, Germany. The study recruited subjects (n = 1851) aged between 25–74 years, and participants were examined between June 2013 and September 2014 at the KORA study center^16^. A whole-body MRI scan (3 Tesla) was incorporated in a sub-study within 400 subjects without known cardiovascular disease, defined as validated/self-reported stroke, myocardial infarction, or revascularization. Further exclusion criteria were contraindications to MRI (e.g. cardiac pacemaker or implantable defibrillator, cerebral aneurysm clip, neural stimulator, any type of ear implant, an ocular foreign body, or any implanted device; pregnant or breast-feeding subjects; claustrophobia) or gadolinium administration (e.g. allergy or serum creatinine level ≥ 1.3 mg/dL). Due to incomplete MRI data and/or inadequate image quality, 22 subjects were excluded^17^. In the current analyses, 378 subjects were included. The KORA FF4 study complied with the Helsinki declaration^18^ on human research, and was approved by the Institutional Research Ethics Board of the Medical Faculty of Ludwig–Maximilian University Munich. Informed consent was obtained from all participants.

### Clinical characteristics

Clinical characteristics including weight, height, body mass index (BMI), body surface area (BSA), smoking history, number of pack-years, prediabetes (normal fasting glucose concentration and a 2-h serum glucose concentration, as determined by oral glucose tolerance test (OGTT), ranging between 140 and 200 mg/dL; and/or an impaired fasting glucose concentration, as defined by fasting glucose levels between 110 and 125 mg/dL, and a normal 2-h serum glucose concentration), diabetes (2-h serum glucose concentration as determined by OGTT that was > 200 mg/dL and/or a fasting glucose level that was > 125 mg/dL), and hypertension (systolic blood pressure < 140 mmHg, diastolic blood pressure < 90 mmHg, or receiving current antihypertensive treatment) were assessed in a standardized fashion as part of the KORA FF4 study as described elsewhere^16,17^.

### Pulmonary function test

Pulmonary function tests were performed in line with the American Thoracic Society and European Respiratory Society recommendations^19,20^. Flow-volume curves were acquired using a pneumotachograph-type spirometer (MasterScope, Jaeger, Hoechberg, Germany). Subjects (n = 216) performed at least 3 and up to 8 spirometry maneuvers to obtain a minimum of two acceptable and reproducible parameters. Residual volume was determined within a measurement of lung diffusing capacity using the single-breath technique with subjects performing a maximum of five trials in order to achieve a minimum of two acceptable and reproducible maneuvers with an effective breath-hold time within 10 ± 2 s.

### Whole-body MR imaging

Whole-body MRI scans were performed with a 3-Tesla MRI system (Magnetom Skyra, Siemens AG, Healthcare Sector, Erlangen, Germany)^17^. The protocol comprised sequences covering the entire body (from neck to below hip) for tissue/organ evaluation^17^. For analysis of the lung, a 2-point DIXON T1 sequence was used, which was acquired in submaximal inspiration breath-hold and lasted for 15 s^15,17^. Slice thickness was 3 mm, coronal acquired, including a field of view (FOV) of 488 × 716, a matrix of 256 × 256, a repetition time (TR) of 4.06 ms and an echo time (TE) of 1.26 ms^15^. For analysis of the heart, the cine-steady-state free precession sequence was acquired in a short-axis view with 10 layers and 25 phases^17^.

### MR image analysis for lung volume

As described previously^15,21^, an algorithm was used for the automatic procession of the MR data. Briefly, the axial in-phase sequence provided the basis to process lung segmentation. The established algorithm is based on a “coarse-to-fine” segmentation strategy and included four major steps^21^; (1) lung and trachea segmentation, (2) trachea and main bronchi extraction, (3) lung separation and (4) filling lung cavities and holes. The automatic algorithm was validated against a ground truth, determined by manual identification of the lung boundaries by two independent experts^21^. In KORA, this algorithm was applied, and all segmentation results were visually checked by one reader unaware of the clinical covariates^15^.

### MR-image analysis of cardiac measurements

LV and RV function were evaluated using commercially available software (cvi42; Circle Cardiovascular Imaging, Calgary, Alberta, Canada) by two independent readers. After manually segmenting the lumens for RV end-systoles and end-diastole in each layer, the software calculated automatically the corresponding volumes. The difference between the end-systolic and end-diastolic volumes comprises the parameters for stroke volume and ejection fraction. Detection of LV contours and calculation of LV volumes was processed automatically, and if necessary, corrected manually according to current guidelines^22^. LV myocardial mass was assessed during end-diastole. Normal values were referenced from a recent publication^23^.

**Figure 1:**
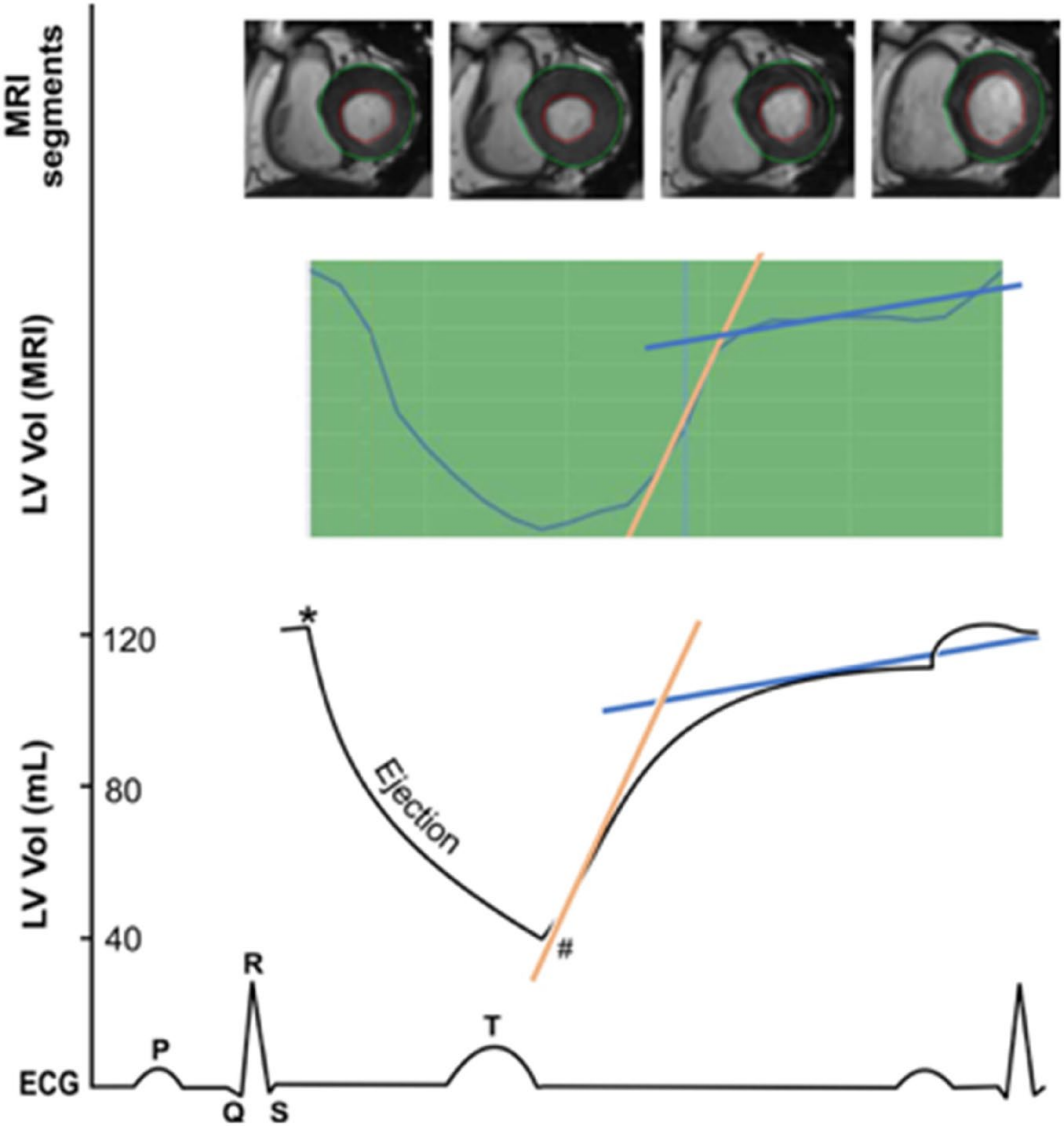
Early and late diastolic filling rate of the left ventricle (slope). In ECG-triggered cine MRI, the filling rates were measured during diastole and assessed by pyHeart, displaying left ventricle (LV) volume versus time curve (middlebox). Example for segmentation of LV in 2-chamber short-axis views above. * = LV end-diastolic volume, # = LV end-systolic volume, yellow line = early diastolic filling rate, blue line = late diastolic filling rate.

Furthermore, filling and ejection rates for LV were quantified using pyHeart, dedicated in-house software displaying LV time-volume-curves (Fig. 1). Peak gradients were anticipated during systolic ejection as well as early LV filling, which is mainly a passive process, and late LV filling, which is driven by atrial contraction^11^.

### Statistical analysis

Clinical characteristics and MRI parameters of cardiac and lung structure and function were presented as arithmetic means with standard deviation (SD) for continuous variables or as counts and percentages for categorical variables. We compared the characteristic differences among tertials of the lung volumes by one-way ANOVA or chi^2^-test. Using linear regression models, cardiac MRI parameters as exposure variables were assessed in relation to FEV1, FVC, residual volume and MRI-derived total lung volume as outcomes. First, in the adjusted model we included BSA. Second, the fully adjusted model included adjustments for age, sex, BSA, and smoking status, providing β-coefficients with 95% confidence intervals (CI). A p-value of less than 0.05 was considered statistically significant with regard to all analyses. Statistical analyses were performed using the Stata 14.1 software package (Stata Corporation, College Station, TX, U.S.A.).

### Ethics approval and consent to participate

The study design was approved by the Institutional Research Ethics Board of the Medical Faculty of Ludwig-Maximilian University Munich. Written informed consent was obtained from all study participants.

## Results

Table 1 presents the clinical characteristics of the study population with a mean age of 56.3 ± 9.2 years and 57% male subjects. 44% of subjects were former smokers, 20% were current smokers, and 36% never smokers. Due to inclusion and exclusion criteria, clinical history for cardiovascular disease was not present.

**Table 1:** Characteristics of the study population.

		All	MRI-derived total lung volume			p
			Low (1.74–3.44L)	Medium (3.45–4.35L)	High (4.36–8.32L)	
n		378	126	126	126	
Age, (years)		56.3 ± 9.2	56.4 ± 9.0	56.1 ± 9.4	56.5 ± 9.2	0.93
Male		217 (57.4%)	34 (27%)	70 (56.6%)	113 (89.7%)	< 0.001
Height, (cm)		171.6 ± 9.7	165.5 ± 8.5	171.9 ± 9.2	177.4 ± 7.3	< 0.001
Weight, (kg)		82.6 ± 16.3	79.3 ± 16.0	82.8 ± 18.2	85.7 ± 13.8	0.01
BMI, (kg/m^2^)		28.0 ± 4.8	28.9 ± 5.1	27.9 ± 5.1	27.2 ± 3.9	0.02
Body surface area, (m^2^)		1.9 ± 0.2	1.9 ± 0.2	2.0 ± 0.2	2.0 ± 0.2	< 0.001
**Smoking status**						0.01
Never		136 (36%)	58 (46%)	38 (32.2%)	40 (31.8%)	
Past		166 (43.9%)	48 (38.1%)	67 (52.4%)	52 (42.3%)	
Current		76 (20.1%)	20 (15.9%)	22 (17.5%)	34 (27%)	
Pack years		13.2 ± 19.0	8.6 ± 16.6	13.5 ± 17.8	17.4 ± 21.4	< 0.001
**Diabetes status**						0.17
Normal		230 (60.9%)	83 (65.9%)	81 (64.3%)	66 (52.4%)	
Prediabetes		99 (26.2%)	29 (23%)	28 (22.2%)	42 (33.3%)	
Diabetes		49 (13.0%)	14 (11.1%)	17 (13.5%)	18 (14.3%)	
HbA1c, (%)		5.57 ± 0.73	5.59 ± 0.64	5.55 ± 0.61	5.58 ± 0.91	0.92
Hypertension		127 (33.6%)	41 (32.5%)	46 (35.5%)	40 (31.8%)	0.69
**Cardiac MR measurements**						
LV	Myocardial mass, diastolic (g)	140.8 ± 35.1	127.4 ± 32.4	139.6 ± 34	155.4 ± 33.5	< 0.001
	End-diastolic volume (mL)	129.2 ± 32.9	125.8 ± 30.4	130.4 ± 35	131.3 ± 33.2	0.36
	End-systolic volume (mL)	40.7 ± 18.1	39.2 ± 18.1	40.8 ± 18.1	42.2 ± 18.2	0.42
	Stroke volume (mL)	88.5 ± 20.5	86.6 ± 18.3	89.7 ± 21.2	89.2 ± 21.7	0.45
	Ejection fraction (%)	69.3 ± 8.7	69.7 ± 7.5	69.6 ± 7.4	68.6 ± 8.5	0.47
	Peak ejection rate (mL/s)	354 ± 133	350 ± 119	353 ± 141	360 ± 139	0.82
	Early diastolic filling rate (mL/s)	226 ± 115	237 ± 108	229 ± 122	212 ± 115	0.21
	Late diastolic filling rate (mL/s)	237 ± 140	243 ± 139	246 ± 152	222 ± 128	0.33
RV	End-diastolic volume (mL)	165.5 ± 39.6	156.4 ± 35.2	167 ± 41.3	174.4 ± 40.2	0.002
	End-systolic volume (mL)	79.5 ± 25.8	72.5 ± 23.1	79.4 ± 26.4	87.3 ± 25.9	< 0.001
	Stroke volume (mL)	86.1 ± 19.5	84 ± 17.2	87.7 ± 20	87.2 ± 21	0.35
	Ejection fraction (%)	52.6 ± 7.1	54.3 ± 6.6	53.1 ± 6.7	50.3 ± 7.3	< 0.001
Pulmonary function		216	76	67	73	
FEV1 (L/s)		3.1 ± 0.8	2.7 ± 0.7	3.2 ± 0.7	3.5 ± 0.7	< 0.001
FVC (L)		4.2 ± 1.0	3.5 ± 0.9	4.3 ± 0.9	4.8 ± 0.9	< 0.001
FEV1/FVC (%)		75.0 ± 7.4	77.8 ± 6.3	74.3 ± 6.6	72.7 ± 8.4	< 0.001
Residual volume (L)		2.2 ± 0.4	1.9 ± 0.4	2.2 ± 0.4	2.4 ± 0.3	< 0.001

The subjects were stratified in tertiles (denoted as low, medium and high) of MRI-derived Total Lung Volume. Pulmonary function test (PFT) was available in a subgroup of 216 subjects. The values represent mean ± standard deviation (SD) or frequency along with percentage.

*P* Represents p-value, *BMI* body mass index, *HbA1c* Glycated hemoglobin A1c, *LV* Left ventricle, *RV* right ventricle, *FEV1* Forced expiratory volume in 1 s, *FVC* Forced vital capacity.

Based on PFT, the average residual volume was 2.2 ± 0.4 L while FEV1 was 3.1 ± 0.8 L, and FVC was 4.2 ± 1.0 L (Table 1). Total lung volume derived from whole-body MRI was 4.0 ± 1.1 L; overall the right lung volume was larger than the left lung volume at 2.2 ± 0.6 L and 1.8 ± 0.5 L, respectively (p < 0.001). Cardiac parameters were within expected ranges (Table 1). Only 9 subjects had an abnormally increased LV end-diastolic volume. Regarding the RV, 17 subjects had an abnormally increased end-diastolic volume; there was some overlap between LV and RV increased end-diastolic volume (n = 6). Based on time-volume curves (Fig. 1), the LV peak ejection rate was 354.9 ± 133 mL/s, while the early and late diastolic filling rates were on average similar (226 ± 115 and 237 ± 140 mL/s, respectively; Table 1). The correlation between early and late diastolic filling rate was 0.25 (p < 0.001) and both parameters correlated with increased LV end-diastolic volume (r = 0.68, p < 0.001; r = 0.29, p < 0.001, respectively), and also with RV end-diastolic volume (r = 0.60, p < 0.001; r = 0.17, p < 0.001, respectively). No reference values were available for the assessment of the time-volume curves.

### Association between cardiac parameters and pulmonary function

**Figure 2:**
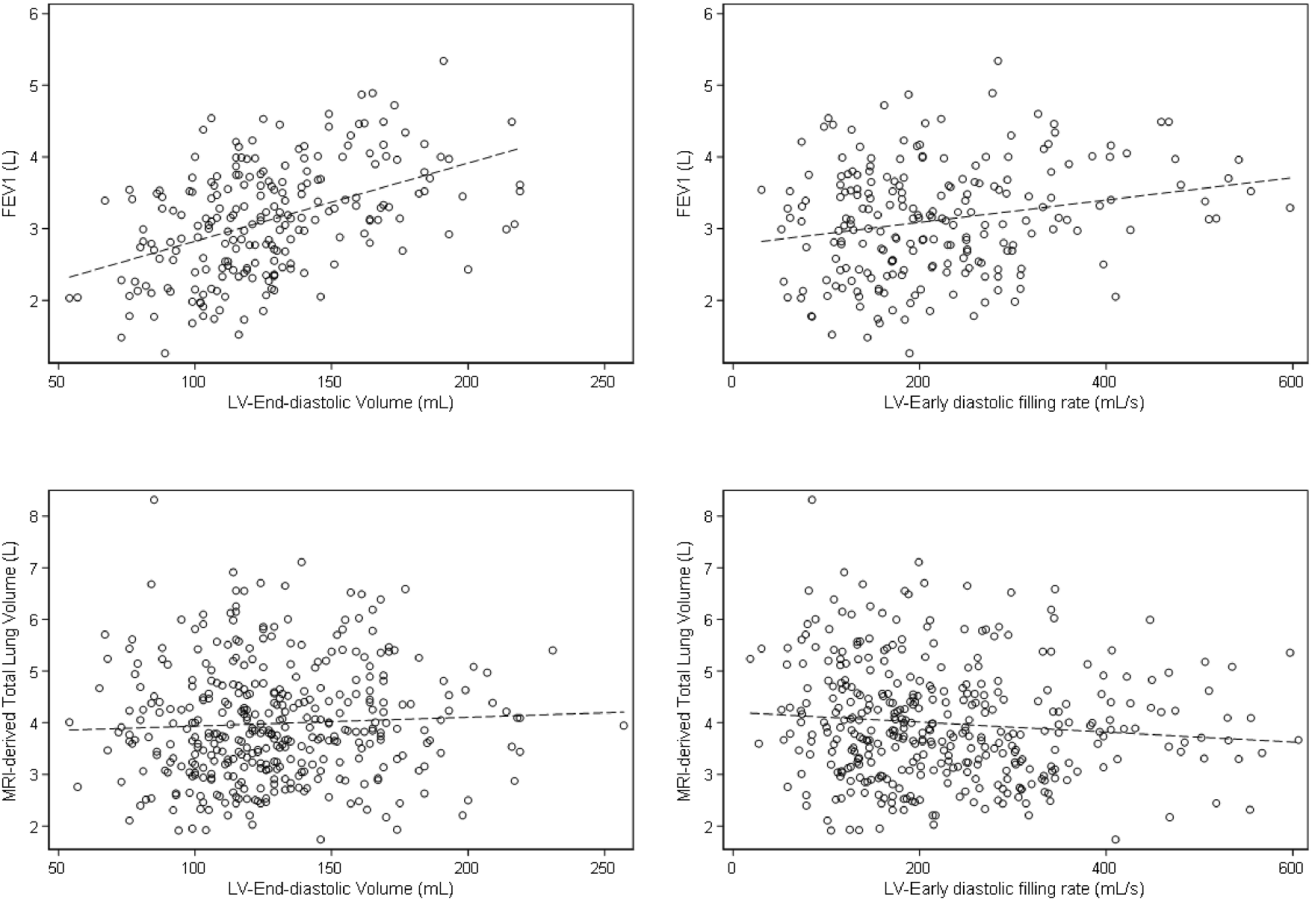
Exemplary plots of the relation of left ventricle parameters with FEV1 and MRI-derived total lung volume (unadjusted). For each association, a regression line (dashed line) is depicted. *FEV1* forced expiratory volume in the first second, *LV* left ventricle.

In the BSA-adjusted model, LV cardiac parameters were associated with FEV1, except for ejection fraction, which was inversely associated, and late diastolic filling rate (non-significant). In a fully adjusted model, cardiac parameters remained associated, except for myocardial mass, ejection fraction and late diastolic filling rate (Table 2). Similarly, in the BSA-adjusted model RV cardiac volumes were associated with FEV1, and after full adjustment only ejection fraction became non-significant. Figure 2 shows the association of LV end-diastolic volume and LV early diastolic filling rate with FEV1 in an unadjusted model. Further, when assessing the relationship between LV cardiac parameters and FVC in the BSA-adjusted model, all parameters were associated with FVC, except for ejection fraction, which was inversely associated, and late diastolic rate (non-significant). In the fully adjusted model, the same parameters remained associated, excluding myocardial mass and ejection fraction, but the association with late diastolic filling rate became significant. Furthermore, RV cardiac parameters were associated with FVC. But after fully-adjustment, ejection fraction became non-significant (Table 2). Moreover, LV end-diastolic volume, end-systolic volume, ejection fraction, peak ejection rate, and early diastolic filling rate were associated with residual volume in the BSA-adjusted model, while again ejection fraction was inversely associated. In a fully adjusted model end-diastolic volume, peak ejection rate, and early diastolic filling rate remained associated, while the late diastolic filling rate turned to be associated as well. While in the BSA-adjusted model RV end-diastolic volume and end-systolic volume were associated with residual volume, after further adjustment most RV parameters were no longer associated, except for stroke volume becoming significantly associated (Table 2). No associations were seen with the Tiffeneau index (data not shown).

**Table 2:** Associations between cardiac MR measurements and pulmonary function.

Per SD		FEV1		FVC		Residual volume	
		Adjusted for BSA	Fully adjusted	Adjusted for BSA	Fully adjusted	Adjusted for BSA	Fully adjusted
		β (95%CI)	β (95%CI)	β (95%CI)	β (95%CI)	β (95%CI)	β (95%CI)
Left ventricle	Myocardial mass	**0.14 (0.01; 0.27)**	0.11 (0.10; 0.12)	**0.20 (0.03; 0.38)**	0.00 (− 0.14; 0.13)	0.03 (− 0.04; 0.11)	− 0.03 (− 0.10; 0.04)
	End-diastolic volume	**0.22 (0.01; 0.27)**	**0.15 (0.07; 0.22)**	**0.28 (0.16; 0.41)**	**0.18 (0.09; 0.28)**	**0.07 (0.01; 0.12)**	**0.05 (0.0; 0.10)**
	End-systolic volume	**0.18 (0.10;0.27)**	**0.10 (0.03;0.17)**	**0.25 (0.14; 0.36)**	**0.13 (0.05; 0.21)**	**0.07 (0.01; 0.10)**	0.03 (− 0.01; 0.08)
	Stroke volume	**0.14 (0.05; 0.24)**	**0.12 (0.05; 0.20)**	**0.17 (0.04; 0.30)**	**0.15 (0.05; 0.24)**	0.05 (− 0.01; 0.10)	0.04 (− 0.01; 0.09)
	Ejection fraction	− **0.12 (**− **0.20; **− **0.04)**	− 0.05 (− 0.11; 0.02)	− **0.19 (**− **0.29; **− **0.08)**	− 0.07 (− 0.15; 0.01)	− **0.05 (**− **0.09; **− **0.00)**	− 0.02 (− 0.06; 0.02)
	Peak ejection rate	**0.17 (0.26; 0.08)**	**0.11(0.18; 0.04)**	**0.25 (0.37; 0.14)**	**0.17 (0.26; 0.09)**	**0.09 (0.13; 0.04)**	**0.07 (0.11; 0.03)**
	Early diastolic filling rate	**0.17 (0.08; 0.26)**	**0.10 (0.03; 0.17)**	**0.25 (0.13; 0.37)**	**0.15 (0.06; 0.24)**	**0.06 (0.01; 0.11)**	**0.05 (0.01; 0.10)**
	Late diastolic filling rate	0.00 (− 0.09; 0.08)	0.03 (− 0.03; 0.10)	0.03 (− 0.08; 0.14)	**0.09 (0.01; 0.17)**	0.03 (− 0.01; 0.08)	**0.05 (0.01; 0.09)**
Right ventricle	End-diastolic volume	**0.29 (0.19; 0.39)**	**0.17 (0.08; 0.25)**	**0.37 (0.24; 0.50)**	**0.19 (0.08; 0.29)**	**0.09 (0.03; 0.14)**	0.05 (− 0.01; 0.10)
	End-systolic volume	**0.28 (0.18; 0.38)**	**0.12 (0.03; 0.21)**	**0.38 (0.25; 0.51)**	**0.14 (0.03; 0.25)**	**0.09 (0.03; 0.15)**	0.03 (− 0.03; 0.09)
	Stroke volume	**0.19 (0.10; 0.29)**	**0.16 (0.08; 0.23)**	**0.21 (0.08; 0.34)**	**0.17 (0.08; 0.27)**	0.05 (− 0.01; 0.11)	**0.05 (0.00; 0.10)**
	Ejection fraction	− **0.11 (**− **0.21; **− **0.02)**	0.03 (− 0.16; 0.11)	− **0.20 (**− **0.33; **− **0.08)**	0.02 (− 0.09; 0.12)	− 0.05 (− 0.10; 0.01)	0.02 (− 0.04; 0.07)

The beta estimate given with a 95% confidence interval represents the association size between cardiac measurements and pulmonary function tests. The fully adjusted model includes the following covariates: age, sex, BSA, and smoking status.

*BSA* Body Surface Area, *CI* 95% confidence interval, *FEV1* forced expiratory volume in the first second, *FVC* forced vital capacity, *SD* standard deviation. Bold font indicates statistical significance (P < 0.05).

### Association between cardiac parameters and MRI-based lung volume

In the BSA-adjusted model, we found an association for LV myocardial mass (β = 0.27, p < 0.01) and an inverse association for LV stroke volume (β = − 0.15, p = 0.02), and early-diastolic filling rate (β = − 0.13, p < 0.02) with MRI-based lung volumes, whereas in the fully-adjusted model stroke volume (β = − 0.14, p = 0.01) and early-diastolic filling rate (β = − 0.11, p < 0.04) remained inversely associated with lung volume, while the association with end-diastolic volume (β = − 0.14, p = 0.01) became significant (Table 3 and Fig. 2). When assessing the association of RV cardiac parameters with lung volume, in the BSA-adjusted model only ejection fraction was inversely associated. However, with the additional fully adjusted model, ejection fraction was no longer associated with lung volume, while stroke volume (β = − 0.11, p = 0.01) became inversely associated with lung volume (Table 3).

**Table 3:** Relation between cardiac measurements and MRI-derived total lung volume.

Per SD		MRI-derived total lung volume	
		Adjusted for BSA	Fully adjusted
		β (95%CI)	β (95%CI)
Left ventricle	Myocardial mass diastolic	**0.27 (0.12; 0.41)**	0.03 (− 0.11; 0.17)
	End-diastolic volume	− 0.11 (− 0.22; 0.01)	− **0.14 (**− **0.25; **− **0.04)**
	End-systolic volume	− 0.02 (− 0.14; 0.09)	− 0.09 (− 0.19; 0.02)
	Stroke volume	− **0.15 (**− **0.26; **− **0.03)**	− **0.14 (**− **0.25; **− **0.03)**
	Ejection fraction	− 0.06 (− 0.18; 0.05)	0.02 (− 0.09; 0.12)
	Peak ejection rate	− 0.02 (− 0.13; 0.08)	− 0.04 (− 0.14; 0.06)
	Early diastolic filling rate	− **0.13 (**− **0.23; **− **0.02)**	− **0.11 (**− **0.21; **− **0.01)**
	Late diastolic filling rate	− 0.09 (− 0.2; 0.02)	− 0.09 (− 0.19; 0.00)
Right ventricle	End-diastolic volume	0.02 (− 0.11; 0.15)	− 0.08 (− 0.2; 0.04)
	End-systolic volume	0.12 (− 0.01; 0.24)	− 0.02 (− 0.14; 0.09)
	Stroke volume	− 0.11 (− 0.23; 0.01)	− **0.11 (**− **0.22; 0.00)**
	Ejection fraction	− **0.21 (**− **0.32; **− **0.09)**	− 0.07 (− 0.18; 0.03)

The beta estimate given with a 95% confidence interval represents the association size between cardiac measurements and MRI derived total lung volume. The fully-adjusted model includes the following covariates: age, sex, BSA, and smoking status.

*BSA* body surface area. Bold font indicates statistical significance (P < 0.05).

## Discussion

In this sample of subjects without clinical history of cardiovascular disease drawn from a general population, we found that several MRI-based cardiac parameters were associated with lung volumes derived from pulmonary function tests. Interestingly, MRI-based cardiac parameters were inversely associated with MRI-based lung volumes, as derived during inspiration. We observed a notable relationship of FEV1, FVC, and residual volume determined by PFT as well as MRI-based lung volumes with selected cardiac measures.

Firstly, we observed an association between end-diastolic and end-systolic volumes, and stroke volume for both LV and RV with FEV1 and FVC, as well as with peak ejection rate, and early diastolic filling rate; additionally, late diastolic filling rate showed an association with FVC. No associations were seen between cardiac parameters and the Tiffeneau index. Secondly, there were significant relationships between LV end-diastolic volume, RV stroke volume, peak ejection rate, and early and late diastolic filling rate, with residual volume. For PFT, our results are consistent with the recent findings from Thomson et al. who showed, using the UK Biobank data, that lower FEV1 and FVC were associated with smaller LV end-diastolic, end-systolic, and stroke volumes, as well as RV end-diastolic, end-systolic, and stroke volumes^13^. Like the UK Biobank, our cohort included only subjects with no history of cardiovascular disease. Comparing the effect size, the estimates in our cohort were significantly larger compared to those in the UK Biobank study. For example, Thomson et al. reported a change of LV end-diastolic volume of − 5.69 mL per SD in FVC, while we observed a change of − 12.51 mL per SD in FVC (one SD of FVC was 1.039L in our cohort). Thomson et al. also observed a change of RV end-diastolic volume of − 5.84 mL per SD in FVC, while we observed a change of − 12.56 ml per SD in FVC, plausibly due to higher body weights in our cohort. However, comparison with the actual PFT parameters was not possible, as these were not reported in the recent publication of the UK Biobank study. Our study adds to the findings of the UK Biobank by showing that FEV1 and FVC may not only affect end-diastolic volumes, but also the diastolic filling rates, which has not been reported so far. We also observed a correlation between the late diastolic filling rate and residual volume, suggesting the impact of residual volume on increased left atrial filling pressures and left atrial contractile function. To our knowledge, this study is the first to describe an inverse association of lung volume with the early diastolic filling rate derived from whole-body MR scans.

With respect to the lung volume derived from whole-body MRI, stroke volume was inversely associated for both LV and RV. For the LV, end-diastolic volume was inversely related; the early, but not the late diastolic filling rate, was inversely related to MRI-based lung volume. Overall, we observed a significant inverse association between lung assessment by MRI and cardiac parameters, in contrast with lung assessment by PFT.

MRI-derived lung volume, as acquired during an inspiration phase, shows a good correlation with total lung capacity (TLC), and residual volume (each r = 0.57)^15^. Although, MR imaging seem to be not the most intuitive technique for lung imaging (low contrast, long acquisition time, etc.), often no other imaging modality is available, particular in large cohort studies including healthy volunteers. Thus, more important is to understand the value of MR lung imaging in correlation to established techniques such as PFT. Previous data also demonstrated also an independent association between MR lung volumes and obstructive ventilatory impairment, based on PFT measurements (Tiffeneau index) and clinical presentation^15^. Whereas the Tiffeneau index is the established parameter to define bronchial obstruction, residual volume can be an indicator of hyperinflation in obstructive lung diseases, and has a unique prognostic value in COPD patients^24^. Nevertheless, we know that PFT has also some limitation and may not always capture the individual disease state.

However, the inverse association between cardiac parameters and MRI-based lung volume contrasted with the positive associations between cardiac parameters and PFT. More specifically, in subjects with both LV end-diastolic volume and early LV diastolic filling decrease, we observed an increased MRI-based lung volume. Again, both cardiac parameters increase with increased residual volume (similarly for FEV1 and FVC). Therefore, this finding may indicate, that pathophysiological characteristics not captured by PFT potentially can be further evaluated through MRI-based assessment of lung volumes. Several mechanisms could explain the contradictory findings between PFT and imaging-based lung assessment in association with cardiac parameters. Breathing mechanics are a likely explanation. PFTs follow a standardized procedure to evaluate lung function and obstructive pulmonary disease, while subjects examined by MRI are often not required to conform to any specific breathing regimen. Additionally, altered breathing patterns, while performing PFT, may occur in subjects with subclinical emphysema. Furthermore, the body posture during measurement should be considered. Subjects are examined in an upright sitting position during PFT, while MRI is performed in supine position. Differences in body position can cause substantial changes in observed lung volumes, including functional residual capacity and residual volume. Moreover, mechanical pressures of the thorax mechanics may be differently affected by body posture in subjects depending on the amount of abdominal fat. Still, imaging can provide pathophysiological insights beyond PFTs for the assessment of ventilatory impairment and lung diseases.

Our findings are supported by previous evidence, as an inverse association between clinically diagnosed severe emphysema and end-diastolic volume for LV measured by cine MRI was described earlier, despite the relatively small number of patients with emphysema (n = 24)^6^. Our results also showed early LV filling impairment and low stroke volumes for both LV and RV, with no change in ejection fraction, in association with lung volume. This is consistent with previous studies where lung volumes were determined by CT and related cine MRI parameters^7,8^, or ECG-gated CT angiography^25^. Also, peak LV filling rates in early and late diastole can be derived from rates of change in chamber volume—a technique made possible by the high spatial resolution of cine MRI (Fig. 1). The peak early and late filling rate in LV are sensitive markers and can indicate early subclinical diastolic dysfunction^26^. However, measuring these volume-derived indices can be a time-consuming process even with advanced software, and therefore impractical in routine clinical care^27^. LV diastolic function indices derived from whole-body MRI may have a future role in screening for early subclinical diastolic, especially if coupled with machine learning techniques.

One strength of our study is the use of an advanced MRI technology 3-Tesla generation, which includes the most advanced imaging modality to-date with well-defined imaging protocol, image processing, and detailed information on the health condition of the study population. Previous studies quantified the lung volumes using CT, which contains radiation-exposure. The lung volume assessment using MRI provides an alternative radiation-free method for large-scale imaging studies. However, our study contains limitations which should be considered. Firstly, because our study included only MRI scans, no comparable lung volume data were generated from CT scans. So far, there are no studies that have shown this direct comparison in the same subjects. However, we could show in our previous study that there is a very good correlation between the TLC from PFT^15^. Secondly, our study is a cross-sectional analysis design and therefore does not allow to assess relationships between alterations in pulmonary and cardiac parameters over time. Thirdly, although our sample size included about 400 subjects, this number is relatively small due to the laborious nature of image processing of whole-body MRI assessment. However, the findings observed in our sample may represent important information for hypothesis generation. Finally, in our sample, only Caucasian participants with no history of cardiovascular disease were included, which may limit the generalizability of our findings.

## Conclusion

Subclinical cardiac impairment was associated with decreasing FEV1, FVC, and residual volume. Cardiac parameters decreased with increasing MRI-based lung volume contrasting the results of PFTs. This suggests that MRI-based lung volume may provide additional information on pathophysiological characteristics beyond PFTs.

## References

[1] Murphy SL, Xu J, Kochanek KD, Arias E (2018). Mortality in the United States, 2017. NCHS Data Brief.

[2] Collaborators GBDCRD (2017). Global, regional, and national deaths, prevalence, disability-adjusted life years, and years lived with disability for chronic obstructive pulmonary disease and asthma, 1990–2015: A systematic analysis for the Global Burden of Disease Study 2015. Lancet Respir. Med..

[3] Chatila WM, Thomashow BM, Minai OA, Criner GJ, Make BJ (2008). Comorbidities in chronic obstructive pulmonary disease. Proc. Am. Thorac. Soc..

[4] Ford ES, Murphy LB, Khavjou O, Giles WH, Holt JB, Croft JB (2015). Total and state-specific medical and absenteeism costs of COPD among adults aged >/= 18 years in the United States for 2010 and projections through 2020. Chest.

[5] Watz H (2010). Decreasing cardiac chamber sizes and associated heart dysfunction in COPD: Role of hyperinflation. Chest.

[6] Jorgensen K, Muller MF, Nel J, Upton RN, Houltz E, Ricksten SE (2007). Reduced intrathoracic blood volume and left and right ventricular dimensions in patients with severe emphysema: An MRI study. Chest.

[7] Barr RG (2010). Percent emphysema, airflow obstruction, and impaired left ventricular filling. N. Engl. J. Med..

[8] Grau M (2013). Percent emphysema and right ventricular structure and function: The multi-ethnic study of atherosclerosis-lung and multi-ethnic study of atherosclerosis-right ventricle studies. Chest.

[9] Demedts IK, Demoor T, Bracke KR, Joos GF, Brusselle GG (2006). Role of apoptosis in the pathogenesis of COPD and pulmonary emphysema. Respir Res..

[10] Barr RG (2007). Impaired flow-mediated dilation is associated with low pulmonary function and emphysema in ex-smokers: The Emphysema and Cancer Action Project (EMCAP) Study. Am. J. Respir. Crit. Care Med..

[11] Caudron J, Fares J, Bauer F, Dacher JN (2011). Evaluation of left ventricular diastolic function with cardiac MR imaging. Radiographics: A review publication of the Radiological Society of North America. Inc.

[12] Malerba M (2011). Sub-clinical left ventricular diastolic dysfunction in early stage of chronic obstructive pulmonary disease. J. Biol. Regul. Homeost. Agents.

[13] Thomson RJ (2018). Variation in lung function and alterations in cardiac structure and function-Analysis of the UK Biobank cardiovascular magnetic resonance imaging substudy. PLoS ONE.

[14] Schlett CL (2016). Population-based imaging and radiomics: Rationale and perspective of the German National cohort MRI study. Rofo..

[15] Mueller J (2019). Automated MR-based lung volume segmentation in population-based whole-body MR imaging: Correlation with clinical characteristics, pulmonary function testing and obstructive lung disease. Eur. Radiol..

[16] Holle R, Happich M, Lowel H, Wichmann HE, Group MKS (2005). KORA–a research platform for population based health research. Gesundheitswesen.

[17] Bamberg F (2017). Subclinical disease burden as assessed by whole-body MRI in subjects with prediabetes, subjects with diabetes, and normal control subjects from the general population: The KORA-MRI study. Diabetes.

[18] World Medical Association Declaration of Helsinki (2013). ethical principles for medical research involving human subjects. JAMA.

[19] Miller MR (2005). Standardisation of spirometry. Eur. Respir. J..

[20] Macintyre N (2005). Standardisation of the single-breath determination of carbon monoxide uptake in the lung. Eur. Respir. J..

[21] Ivanovska T (2012). A fast and accurate automatic lung segmentation and volumetry method for MR data used in epidemiological studies. Comput. Med. Imaging Graph..

[22] Grover S, Leong DP, Selvanayagam JB (2011). Evaluation of left ventricular function using cardiac magnetic resonance imaging. J. Nucl. Cardiol..

[23] Petersen SE (2017). Reference ranges for cardiac structure and function using cardiovascular magnetic resonance (CMR) in Caucasians from the UK Biobank population cohort. J. Cardiovasc. Magn. Reson..

[24] Miller MR (2005). General considerations for lung function testing. Eur. Respir. J..

[25] Huang Y-S, Hsu H-H, Chen J-Y, Tai M-H, Jaw F-S, Chang Y-C (2014). Quantitative computed tomography of pulmonary emphysema and ventricular function in chronic obstructive pulmonary disease patients with pulmonary hypertension. Korean J. Radiol..

[26] Kudelka AM, Turner DA, Liebson PR, Macioch JE, Wang JZ, Barron JT (1997). Comparison of cine magnetic resonance imaging and Doppler echocardiography for evaluation of left ventricular diastolic function. Am. J. Cardiol..

[27] Leong DP, De Pasquale CG, Selvanayagam JB (2010). Heart failure with normal ejection fraction: The complementary roles of echocardiography and CMR imaging. JACC Cardiovasc. Imaging.

